# Adenovirus vector and mRNA vaccines: Mechanisms regulating their immunogenicity

**DOI:** 10.1002/eji.202250022

**Published:** 2022-11-20

**Authors:** Nicholas M. Provine, Paul Klenerman

**Affiliations:** ^1^ Translational Gastroenterology Unit Nuffield Department of Medicine University of Oxford Oxford UK; ^2^ Peter Medawar Building for Pathogen Research University of Oxford Oxford UK

**Keywords:** adenovirus vector, mRNA vaccines, COVID‐19, innate response, cellular memory

## Abstract

Replication‐incompetent adenovirus (Ad) vector and mRNA‐lipid nanoparticle (LNP) constructs represent two modular vaccine platforms that have attracted substantial interest over the past two decades. Due to the COVID‐19 pandemic and the rapid development of multiple successful vaccines based on these technologies, there is now clear real‐world evidence of the utility and efficacy of these platforms. Considerable optimization and refinement efforts underpin the successful application of these technologies. Despite this, our understanding of the specific pathways and processes engaged by these vaccines to stimulate the immune response remains incomplete. This review will synthesize our current knowledge of the specific mechanisms by which CD8^+^ T cell and antibody responses are induced by each of these vaccine platforms, and how this can be impacted by specific vaccine construction techniques. Key gaps in our knowledge are also highlighted, which can hopefully focus future studies.

## Graphical abstract

Considering the success of adenovirus vector and mRNA vaccines against COVID‐19 an understanding of their underlying immunology is of interest. We discuss current knowledge of the processes involved in the immunogenicity of these platforms with respect to target cell populations, antigen presentation, innate immune activation, and mechanisms of immune priming.

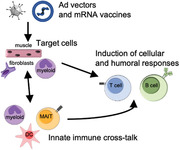

## Introduction

The COVID‐19 pandemic has seen the first widespread usage of two new vaccine technologies: replication‐incompetent adenovirus (Ad) vectors and mRNA vaccines. This pandemic has brought to market, in record time, four separate Ad vector‐based vaccines (Janssen's Ad26.COV2.S, Oxford‐AstraZeneca's ChAdOx1 nCoV‐19, CanSino's Ad5‐nCOV, and The Gamaleya Research Institute's Gam‐COVID‐Vac) and two mRNA vaccines (Moderna's mRNA‐1273 and Pfizer‐BioNTech's BNT162b2) [1]. An additional Ad vector‐based vaccine against Ebola virus (Janssen's Ad26.ZEBOV) was licensed in 2020 [2], but has not needed widespread use. While these two technologies have only recently emerged as clinical products, they have been under development for some time. The first animal testing of a replication‐incompetent Ad vector as a vaccine platform was reported in 1996 [3] and the first mRNA vaccine in 1993 [4]. Considerable effort has gone into the development and refinement of these vaccine platforms. However, despite these efforts, and their recently proven clinical utility, there remains a major gap in our understanding how these vaccines interact with the immune system to induce robust cellular and humoral immune responses. This review will discuss our current understanding of the fundamental immunology of these technologies and highlight gaps in our knowledge.

### Substantial heterogeneity in vaccine construction technique complicates generalizability of findings

As Ad vectors and mRNA vaccines have been under development for several decades, this has led to considerable diversity in the specific construction techniques of these vaccines.

Although there is some variation in construction technique, the standard approach to convert adenovirus into replication‐incompetent vector involves the deletion of the E1 and E3 genes [5]. These deletions render the vector replication‐incompetent (E1 deletion) and removes the E3 genes, which have immunomodulatory capacity [6, 7, 8]. These deletions also provide spare coding capacity within the viral genome for insertion of the transgene product of interest. Human adenovirus serotype 5 (Ad5) was the most widely used Ad vector for many early studies including human gene therapy trials and phase II HIV vaccine trials [9, 10, 11]. However, concerns about the safety profile of Ad5 vectors in populations with high HIV seroprevalence have dampened enthusiasm in some circles for vaccines based on this specific viral backbone [12]. This, combined with high seroprevalence of Ad5, led to the development of a number of alternative vaccine vectors based on either low seroprevalence human adenoviruses (e.g. adenovirus serotype 26) or non‐human primate‐derived viruses (e.g. ChAdOx1) [13, 14, 15]. Given this history, most studies have used Ad5 for investigating the fundamental immunology of Ad vectors. However, emerging data suggest that distinct serotypes of Ad vectors have very different biology (reviewed in [16]). We will highlight areas where differences in the immunology of the distinct vector serotypes is known.

mRNA vaccines display an even wider variation in construction techniques, as both the nature of the RNA molecule itself and the encapsulating lipid can be specifically engineered (reviewed in [17]). While both currently licensed mRNA vaccines are in the class of nucleoside‐modified mRNA vaccines, they have considerable differences in the nature of their encapsulating lipid nano‐particles (LNPs) [18]. Self‐amplifying RNA (saRNA) and unmodified mRNA vaccines have also progressed to human testing [19, 20], and have long development histories. Given this diversity, it is even more difficult with mRNA vaccines to determine how the immunology of one construct might inform the biology of another. Platform‐specific findings will be discussed.

## Adenovirus Vectors

### Regulation of adenovirus vector‐induced CD8^+^ T cell responses

#### Antigen localization and CD8^+^ T cell responses

**Figure 1:**
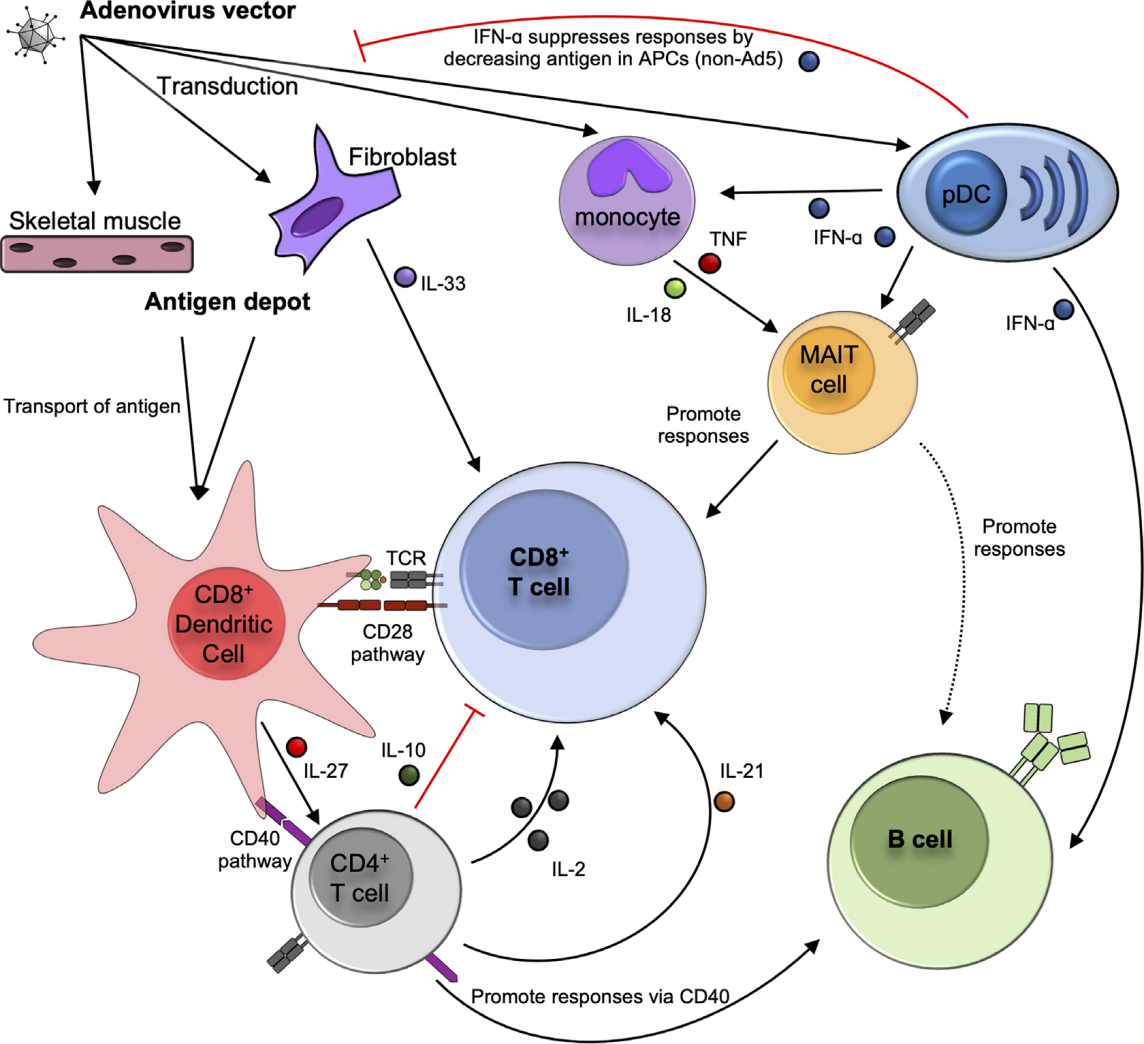
**Pathways known to promote or inhibit the induction of cellular and humoral immune responses following adenovirus vector immunization**. Key pathways and immune processes identified by mouse studies are shown in solid lines. Black arrows denote processes shown to promote immune responses, while red lines denote inhibition. Dashed lines indicate where correlative data in humans or non‐human primates suggests an interaction between cell types. Where cells have been shown to interact, but molecular mechanisms have not been determined, this is noted. Not all pathways have been examined for all different major serotypes of Ad vectors, so the presented model is a synthesis of studies using the different vectors. Known cases of vector‐specific differences are noted in the model. See text for references for indicated pathways.

The first step in priming of a T cell response is processing and presentation of an antigen by professional antigen‐presenting cells (APCs) within the draining lymph node (LN). As would be expected, CD11c^+^ dendritic cells (DCs) are the primary APC following Ad vector immunization [21]. However, several studies have shown that following intramuscular immunization, most antigen is present in the muscle and that the amount of antigen directly detectable in the draining LN is markedly lower (Fig. 1) [22, 23, 24]. This is especially true for studies examining vectors other than Ad5, where trafficking to the LN appears particularly inefficient [22, 25]. Reconciling these points, it appears that CD8^+^ DCs are the critical APC population for the priming of CD8^+^ T cell responses to all serotypes of Ad vectors tested (Fig. 1) [25, 26, 27]. This DC subset has the unique capacity to endocytose exogenous material and through a retrograde transport process load this material into the MHC class I antigen presentation pathway, in a process termed “cross presentation” [28]. Cross presentation allows for the priming of naïve CD8^+^ T cells even if DCs are not directly infected by a pathogen. Development of this population is dependent on the transcription factor *Batf3* [29], so *Batf3^−/−^* mice, as used in the mentioned studies, lack the capacity for cross presentation.

Provision of Signal 2 from DCs to naïve T cells during priming is the second critical step [30], which usually takes the form of DC‐expressed CD80 and CD86 interacting with CD28 on T cells. Consistent with this, *Cd80^−/−^Cd86^−/−^* mice have impaired CD8^+^ T cell responses after Ad5 immunization, but for reasons that remain to be elucidated *Cd28^−/−^* mice have a different phenotype: delayed but not fully impaired CD8^+^ T cell responses (Fig. 1) [27, 31]. TNF receptor super‐family (TNFRSF) members have a broad role in regulating the functionality of CD8^+^ T cells [32]. Two TNFRSF genes, OX‐40 and 4‐1BB, have opposite roles in modulating the primary CD8^+^ T cell responses to Ad5 [33]. The absence of OX‐40 impaired the expansion and effector functionality of the CD8^+^ T cell response, and this corresponded with an increased expression of memory‐associated markers (CD27 and CD62L). By contrast, absence of 4‐1BB resulted in a hyper effector response of greater magnitude and functionality.

#### Persistent antigen and memory inflation

The typical kinetics of a T cell response can be divided into three phases: (1) the rapid expansion of the primary effector response, (2) followed by contraction of the responding population upon resolution of infection, and (3) ultimately stable maintenance of a long‐lived memory population [34]. By contrast, “memory inflation” is a phenomenon where the T cell response does not contract after the initial acute effector response and instead an expanded T cell population is maintained long‐term (recently reviewed in [35]). While many of the details of the molecular processes driving memory inflation are outside the scope of this review, the key prerequisite for memory inflation is low‐level persistence of antigen [35], as first described in the context of T cell responses to herpes viruses [36]. While direct transduction of the draining LN appears inefficient following Ad vector immunization, antigen production has been reported for weeks following intramuscular and intravenous immunization with Ad5, Ad26, and ChAdOx1 vectors (Fig. 1) [22, 23, 37–39]. An elegant study using an Ad5 vector where transgene expression could be silenced by administration of doxycycline [40] found that persistent antigen expression was required for the maintenance of OVA‐specific CD8^+^ T cells at a high frequency. Intravenous administration of an Ad5 vector expressing a β‐galactosidase transgene is now well‐established as a model of memory inflation [38].

It is unclear to what extent the memory inflation effector program reflects the standard phenotype and function of T cells induced by Ad vector immunization, as the phenomenon has not been systematically studied outside of Ad5‐derived constructs. However, comparison of Ad5‐induced responses in mice and Ad6‐induced (another species C vector) responses in humans found notable similarities in the profiles of the induced CD8^+^ T cell responses [41]. Furthermore, several studies examining the kinetics of Ad5‐induced CD8^+^ T cell responses against a variety of model antigens observed responses that remained stable over the study period [40, 42–44], suggesting that induction of ‘inflating’ responses may be a default trait of Ad5‐derived vectors. Using bone marrow chimeras, non‐hematopoietic cells were shown to have a role in Ad5‐induced CD8^+^ T cell responses [45]. A recent study has mechanistically explained this observation, as Cupovic *et al.* identified CCL19‐expressing fibroblasts as necessary for Ad5‐induced memory inflation in a process dependent on the production of IL‐33 (Fig. 1) [26]. In this study, a ChAdOx1 vector was shown to be as efficient as an Ad5 vector at transducing fibroblasts opening the possibility that other vectors are equally capable of inducing memory inflation, although examination of ChAdOx1‐induced T cell responses was not performed.

In addition to maintenance of antigen‐specific CD8^+^ T cells at high frequencies, another defining trait of inflationary responses is the long‐term persistence of “effector‐like” phenotype and functionality. Following acutely resolved viral infection, such as the prototypic LCMV Armstrong, quiescent memory CD8^+^ T cells rapidly upregulate CD127 and CD62L and acquire expression of IL‐2 [46, 47]. In contrast, Ad5‐induced CD8^+^ T cell responses are characterized by the slow acquisition of markers of memory CD8^+^ T cells such as CD127 and CD62L and IL‐2 expression, as well as persistent expression of KLRG1 [23, 38, 42, 43, 48–50]. This phenotype is partially dependent on persistent antigen, as evidenced using the doxycycline‐sensitive vector [40], and consistent with the inflationary nature of the response. However, it may be restricted to specific epitopes – dependent at least in part on inter‐epitope competition [51].

In contrast to Ad5, CD8^+^ T cell responses induced by Ad26, Ad35, and Ad48 vectors have accelerated conversion to a memory phenotype [42, 43, 49]. One study found that antigen levels and persistence were lower following intramuscular ChAdOx1 immunization as compared to Ad5 [22], but another study using Ad26 found this vector persisted at higher levels than Ad5 [37]. A recent study found that following intramuscular immunization of mice with the ChAdOx1 nCoV‐19 vaccine, soluble S1 spike was strongly detectable in the serum after 72 hours, but nearly undetectable by seven days [52], suggesting only transient systemic antigen availability. However, another biodistribution study of the same vaccine found detectable vector DNA at the injection site and draining LNs for four weeks following immunization [53], suggesting the possibility of persistent low levels of local antigen. Persistent viral DNA exists in an extrachromosomal form as adenoviruses have no encoded capacity for integration and experimental estimates of integration rates of Ad5 put it at a median frequency of ∼0.01% per transduced cell [54, 55]. None of these studies directly tested the impact of antigen persistence on the T cell responses induced by ChAdOx1 or Ad26. As discussed in later sections, it appears that differences in transgene persistence may be only one reason for the different phenotype and kinetics of the response induced by Ad5 versus alternative vectors. Clearly, further investigation is required to determine if memory inflation is a standard phenotype induced by Ad vector vaccination, or a specific phenomenon induced by Ad5 in the context of certain CD8^+^ T cell epitopes.

#### Regulation of CD8^+^ T cell responses by innate cytokines

After recognizing cognate antigen via the TCR (signal 1) and receiving co‐stimulatory signals via CD28 (signal 2), the third signal required for T cell priming is provided by cytokines [56]. The two prototypic “signal 3” cytokines are IL‐12 and type I interferon. Somewhat surprisingly, the role of IL‐12 in Ad vector‐induced immune responses has only been analyzed in a limited way. One study found IV delivery of Ad5 induced IL‐12 production which activated NK cells and thereby drove CD8^+^ T cell expansion [57]. Interestingly, this response was much stronger in BALB/c mice as compared to C57Bl/6. Consistent with a possible lesser role in C57Bl/6 mice, another study specifically examining CD4^+^ T cell responses in C57Bl/6 mice, found no impact of IL‐12 deficiency on this population following Ad5 immunization [37].

The role of type I interferon has been more extensively examined, and appears to have a vector strain‐specific effect (Fig. 1). Several studies have reported unaltered CD8^+^ T cell responses in either *Ifnar1^−/−^* mice or mice receiving anti‐IFNɑR1 antibodies and immunized with Ad5 vectors [25, 49, 58]. Minimal induction of type I interferons in vitro and in vivo by Ad5, especially compared to vectors derived from alternate serotypes, has been reported [49, 59–62], which likely explains this observation. At least in vitro, poor induction of type I interferon by Ad5 is mechanistically linked with inefficient transduction of plasmacytoid dendritic cells [59, 62, 63]. Another study suggests that direct binding of Ad5 fiber to the cellular protein Gas6 also contributes to dampened IFN signals [64].

By contrast, alternate serotype vectors (i.e. not Ad5; derived from clades B, D, and E) efficiently transduce pDCs, strongly induce type I interferon production, and this can impair transgene‐specific CD8^+^ T cell response depending on the exact vector and dose [25, 49, 59]. Mechanistically the sensors responsible for detection of non‐Ad5 vectors remain unclear. In vitro, absence of TLR9 signaling inhibits type I interferon production [63, 65]. Multiple studies report that while *Myd88^−/−^* mice (the signaling adaptor for TLR9) have impaired vaccine‐induced CD8^+^ T cell responses [21, 66]; this same defect is not seen in *Tlr9^−/−^* mice [21, 65, 66]. Instead, *Sting^−/−^* mice had enhanced CD8^+^ T cell responses, phenocopying the *Ifnar1^−/−^* mice [25]. Thus, further work is required to fully determine in vivo the which aspects of each viral vector's biology are driving type I interferon production.

The other main family of cytokines that has been investigated for a role in priming of CD8^+^ T cell responses are the inflammasome‐derived cytokines IL‐1 and IL‐18. One of the early seminal studies describing how the NLRP3 inflammasome functions identified Ad5 as a potent stimulator in vitro [67]. Encapsulation of viral DNA was necessary to induce maturation and secretion of IL‐1β, as neither empty capsids nor helper‐dependent vectors (which do not incorporate viral DNA [68]) were stimulatory. Subsequent studies refined our mechanistic understanding of the process by demonstrating that endosomal escape of the virion via rupture of the endolysosome was necessary to induce IL‐1β maturation as a temperature‐sensitive mutant that failed to escape the endosome also failed to trigger IL‐1β maturation [69, 70, 71]. Rupture of the endosome releases Cathepsin B into the cytoplasm, which is a potent inflammasome trigger [72]. A caveat of these early in vitro studies is that they were performed at very high multiplicities of infection (MOI) ranging from 10^4^ to 5×10^5^ viral particles (vp) per cell.

Alternate serotype vectors appear more efficient than Ad5 at triggering maturation of IL‐1β and IL‐18, especially at lower MOIs [59, 61]. Mechanistically, the process appears the same as seen with the higher doses of Ad5: Cathepsin B‐mediated activation of the NLRP3 inflammasome, as inhibitors of this pathway efficiently block this process [59, 60]. The increased stimulatory nature of Ad26 and Ad35 was linked with preferentially trafficking of these vectors to the late endosome, which is enriched for Cathepsin B [73], while Ad5 escaped from the endolysosomal pathway at an earlier stage of trafficking [60]. Thus, the lysis of late endosomes, enriched for Cathepsin B, would provide a stronger trigger of inflammasome activation.

The relative difference in efficiency of Ad5 versus other Ad vectors for triggering inflammasome activation and thus IL‐1β and IL‐18 release may explain why no major role for these proteins has been described in vivo. One study found no alteration in the frequency or functionality of antigen‐specific CD8^+^ or CD4^+^ T cells following Ad5 immunization of *Nlrp3^−/−^* or *Asc^−/−^* mice (lacking the inflammasome signaling adaptor) [21]. A second study found similar results following Ad5, Ad26, or Ad35 immunization of *Il1r1^−/−^* mice, but the kinetics of the response appeared delayed following Ad26 or Ad35 immunization of *Il18ra^−/−^* mice [66]. However, there are limits to interpretation of these studies given the group sizes are small and the responses have not been studied in much detail. Thus, a more systematic investigation of the role of IL‐1β and IL‐18 in the regulation of Ad vector‐induced (both Ad5 and otherwise) CD8^+^ T cell responses is required to fully determine the importance of these cytokines, although these early data would suggest the individual impact is minor.

#### Regulation of CD8^+^ T cell responses by CD4^+^ T cells

The role of several different lymphocyte populations in promoting Ad vector‐induced immunity has been analyzed (Fig. 1). CD4^+^ T cells are commonly termed “T helper cells” due to their ability to enhance (“help”) CD8^+^ T cell and antibody responses. With regards to CD8^+^ T cell responses, CD4^+^ T cell help can be provided at multiple stages of the immune response. CD4^+^ T cell help to CD8^+^ T cells was first described as the process of imparting CD8^+^ T cell responses with enhanced anamnestic potential [74, 75, 76]. This process involves DCs acting as a bridge between CD4^+^ T cells and CD8^+^ T cells, and signaling via CD40 is critical for transmission of this help signal. Absence of CD40 signaling impairs the anamnestic potential of Ad vector‐induced CD8^+^ T cell responses (Fig. 1) [77]. Subsequent work has also demonstrated a role for CD4^+^ T cells in maintaining memory CD8^+^ T cell populations [78, 79, 80, 81], and also in promoting expansion of primary CD8^+^ T cell responses [82, 83, 84, 85]. Although the role for CD4^+^ T cell help is dependent on the specific pathogen/vaccine under investigation, as in some of these experimental systems the primary CD8^+^ T cell responses are unimpaired [81, 86].

In the context of Ad vector vaccination, CD4^+^ T cell help appears to be critical in all three of these settings. The absence of CD4^+^ T cells dramatically impaired the expansion of a primary CD8^+^ T cell response and accelerated its contraction following Ad5 and Ad26 immunization [38, 77, 87, 88]. CD4^+^ T cell help is also required for proper acquisition of effector functions and differentiation of responding CD8^+^ T cells [44]. These responses primed in the absence of CD4^+^ T cells had reduced cytokine production and cytotoxic functionality, increased expression of inhibitory receptors, and a transcriptional program similar to T cell exhaustion. One study found provision of exogenous IL‐2 could partially reverse these defects in unhelped CD8^+^ T cells, but it was not formally proven that a lack of CD4^+^ T cell‐derived IL‐2 explained the impaired responses (Fig. 1) [44]. This phenotype has also been described in the context of cancer [89], suggesting this phenotype may be a general characteristic of “unhelped” CD8^+^ T cells, as opposed to a unique property of Ad vector‐induced responses. CD4^+^ T cells are producers of IL‐21, and absence of IL‐21 impairs the primary Ad vector‐induced CD8^+^ T cell response [90, 91], suggesting another possible mechanism of CD4^+^ T cell help (Fig. 1).

While CD4^+^ T cells have a clear role in promoting Ad vector‐induced CD8^+^ T cell responses, the signals they generate do not always enhance responses. Ad5 vectors are particularly immunogenic in mice with T cell responses plateauing or declining at high doses [37, 48, 92–95]. IL‐27‐induced production of IL‐10 by CD4^+^ T cells is a major driver of these impaired CD8^+^ T cell responses following immunization with high doses of Ad5 (Fig. 1) [37]. That this phenotype is not observed in mice at equivalent doses of Ad26 vector likely contributes to the differences in phenotype between Ad5‐ and Ad26‐induced responses. Thus, CD4^+^ T cells critically positively and negatively regulate Ad vector‐induced CD8^+^ T cell responses.

#### Regulation of CD8^+^ T cell responses by other lymphocyte populations

Beyond CD4^+^ T cells, the role of several other lymphocyte subsets in regulating Ad vector‐induced responses has been examined. Mucosal‐associated invariant T (MAIT) cells are a population of T cells that express a semi‐invariant T cell receptor that recognizes non‐peptide antigens derived from vitamin B biosynthesis pathways (reviewed in [96]). These cells can be activated by cytokines, akin to an NK cell or ILC. We recently demonstrated that this mode of activation is relevant in the context of ChAdOx1 vector immunization, as vector‐induced type I interferon, TNF, and IL‐18 worked in concert to drive MAIT cell activation (Fig. 1) [59]. Activation was observed in response to an array of vectors, excluding species C‐derived vectors (including Ad5), which were poorly stimulatory. Ad vector‐induced MAIT cell activation was associated with increased vaccine immunogenicity in human volunteers, and *Mr1^−/−^* mice, which lack MAIT cells [97], had impaired conventional CD8^+^ T cell responses [59]. CD4^+^ T cell responses were not examined in detail, but appeared to be unimpaired in mice lacking MAIT cells. Mechanistically how MAIT cells enhance CD8^+^ T cell responses remains to be determined, but transcriptional analysis of MAIT cells identified elevated expression of the CXCR3‐binding chemokines (CXCL9/10/11), which suggests a possible role for MAIT cells in recruiting CD8^+^ T cells into the response. Vδ2^+^ γδT cells are a functionally related population of unconventional T cells [98, 99, 100], which are present in humans but not mice [101]. Ad vector immunization activates Vδ2^+^ γδT cells by similar pathways as it does MAIT cells [102], but the lack of a relevant mouse model to study these cells makes it difficult to assess their functional role.

NK cells represent the other highly abundant population of cytokine‐responsive lymphocytes, which can respond to the same stimuli as MAIT cells [99]. One study found that CD8^+^ and CD4^+^ T cell responses induced by Ad5 vaccination were unimpaired in magnitude and phenotype in mice depleted of NK cells [103]. A caveat to interpreting these data is that Ad5 poorly induces the cytokines associated with activation of the innate(‐like) lymphocyte populations [25, 59, 61]. So, the lack of a reported role for NK cells in modulating vaccine‐induced immunity may partially reflect the choice of vector used in this study. An earlier study found that NK cells regulated Ad vector‐induced CD8^+^ T cell responses in BALB/c, but not C57BL/6, mice due to increased production of IL‐12 by this strain in response to Ad vector stimulation [57]. Further work will be required to fully understand the role of NK cells in Ad vector‐induced immune responses.

### Regulation of adenovirus vector‐induced antibody responses

Despite a recent surge in interest in the ability of Ad vectors to induce protective antibody responses (particularly in the context of SARS‐CoV‐2), these vectors have primarily historically been used with the aim of inducing T cell responses. Thus, the pathways and processes which regulate the induction of antibody responses remain poorly studied.

#### Memory B cell inflation?

As discussed above, Ad5 vectors can induce inflationary CD8^+^ T cell responses. The prototypic inflationary viral infection (murine cytomegalovirus; MCMV) has also been shown to induce inflationary B cell responses, which are characterized by gradually increasing IgG antibody titers that do not wane [104]. Whether such an inflationary antibody response is induced in mice following Ad vector immunization is unknown. One study, using an Ad26 vector, found antibody titers were stable for at least 90 days after a single immunization [105]. Long‐term follow‐up of clinical trials of Ad5‐, Ad26‐, and ChAdOx1‐based vaccines show relatively stable antibody titers for at least 6 months after a single Ad vector dose [106, 107, 108], suggesting such inflationary antibody responses may be relevant in humans as well. This area requires further investigation.

#### Regulation of antibody responses by CD4^+^ T cells

CD4^+^ T cell help is also required for the induction of antibody responses to the Ad vector‐encoded transgene product (Fig. 1) [105]. An earlier study found that the induction of vector‐specific neutralizing antibodies also requires the presence of CD4^+^ T cells [109, 110], consistent with the general need for CD4^+^ T cell help in the induction of antibody responses against protein antigens [111]. Consistent with this general model, CD40 signaling was identified as a critical pathway in the induction of an antibody response (Fig. 1) [31, 105]. Signaling via CD80/CD86 was also necessary for the induction of anti‐vector antibody responses [31], likely due to a need for this pathway in priming CD4^+^ T cells. Depletion of macrophages impaired induction of anti‐vector and anti‐transgene antibody responses, as well as T cell responses [112]. Whether this reflects a direct role for macrophages in B cell priming, CD4^+^ T cell priming, or both, remains to be determined. Surprisingly, if the CD4^+^ T cell population was only transiently depleted at the time of vaccination, then a delayed, but fully functional antibody response was induced, which coincided temporally with the repopulation of the CD4^+^ T cell compartment [105]. As CD4^+^ T cell depletion facilitates transgene persistence [44, 113, 114], it is unclear if these data reflect de novo priming of a naïve B cell population, or a delayed induction of a germinal center (GC) response in previously primed B cells.

#### Regulation of antibody responses by cytokines

In contrast to Ad vector‐induced CD8^+^ T cell responses (discussed above), absence of type I interferon signaling was found to impair GC B cell responses, IgM titers, and IgG titers following vaccination through B cell intrinsic and extrinsic processes (Fig. 1) [115]. Through a series of adoptive transfer experiments, the authors identified type I interferon signaling to DCs to be critical for promoting secretion of IgM, but dispensable for GC responses and antibody class switching. In contrast, interferon signaling to both B cells and CD4^+^ T cells was required for optimal production of IgG. Another study examining the impact of TLR4 deficiency on Ad5‐induced humoral immune responses also identified impaired GC responses, lower transgene‐specific IgG titers, and impaired CD4^+^ T cell responses [116]. Whether this shared phenotype reflects a common pathway around impaired induction of type I interferon in the absence of TLR4 signaling was not explored. These results are striking given other studies have not identified strong production of type I interferon following immunization with Ad5 [49, 117]. Thus, further work is required to reconcile these potentially conflicting results.

#### Regulation of antibody responses by other lymphocyte populations

A recent study found that volunteers who produced stronger neutralizing antibody responses following vaccination with the ChAdOx1 nCoV‐19 vaccine had increased activation of NK cells on day 3 post‐vaccination, as opposed to volunteers who generated weaker antibody responses [118]. Transcriptomic analysis of rhesus macaques immunized with a single dose of Ad26.COV2.S showed a positive association between NK cell activation and plasma cell responses [119]. Whether this reflects a direct role for NK cells in promoting antibody responses following Ad vector immunization, or simply reflects a useful biomarker for vaccine immunogenicity, remains to be determined. MAIT cell activation (assessed by transcriptomics) and type I interferon signatures were also associated with increased memory B cell and antibody responses following Ad26 immunization (Fig. 1) [119]. However, as discussed above, type I interferons appear to have a direct role in promoting B cell responses following vaccination, so this may reflect two distinct phenomena. In a rhesus macaque SIV vaccine model, Ad5 vaccination was found to increase frequencies of MAIT cells in the circulation and BAL (bronchoalveolar lavage fluid), and the frequency of MAIT cells following vaccination correlated with some, but not all, tested measures of transgene antigen‐specific B cell and antibody responses [120]. Several studies have demonstrated some capacity for MAIT cells to promote B cell responses upon TCR‐driven activation [120, 121, 122]. However, being a virus Ad vectors fundamentally cannot provide the cognate TCR ligand for MAIT cells, and Ad5 poorly activates MAIT cells via cytokines [59]. Thus, while it is an intriguing possibility that MAIT cells may promote Ad vector‐induced antibody responses, it is unclear mechanistically how this might occur. Further work is required to provide clear functional links between these lymphocyte populations and modulation of antibody responses.

### Regulation of immune responses by anti‐vector immunity

In addition to inducing immune responses towards the encoded transgene, Ad vector immunization elicits immunity against the viral particle itself. This immunity includes both anti‐vector antibodies and cellular immune responses [109, 123, 124], and both of these responses can independently impair vaccine immunogenicity. Impaired immunogenicity in patients with high levels of anti‐Ad5 antibody responses has been reported in several clinical trials [9, 125]. This is of particular concern in the context of Ad5 as pre‐existing antibody responses against this virus are prevalent in a large fraction of the population from an early age in some parts of the world [126]. In contrast, pre‐existing antibody responses to Ad26 and ChAdOx1 are rare and generally of lower titer in unvaccinated individuals [13, 14, 126, 127]. Mechanistically, pre‐existing anti‐vector immunity impairs vaccine responses by blocking transduction and expression of vaccine products [109]. As most of the antibody response is directed against the hypervariable regions of the hexon capsid subunit, swapping these regions to vector serotypes with low seroprevalence can be an effective strategy to evade pre‐existing immunity [128].

## mRNA vaccines

Unlike the adenovirus vector field, where antibody and T cell responses have historically been studied separately, the investigation of mRNA vaccine cellular and humoral immunogenicity has been performed in a more integrated manner. Thus, the following sections are not divided specifically into regulation of antibody and T cell responses, but instead focus on what is known about how certain processes regulate both arms of adaptive immunity.

### Antigen localization and induction of cellular and humoral immunity

With mRNA vaccines both the lipid nanoparticle (LNP) encapsulating the mRNA and the mRNA itself play a role in regulating the priming of adaptive immune responses. Engineering of the mRNA molecule to be a self‐amplifying (saRNA) construct – where the vaccine encodes an RNA‐dependent RNA‐polymerase (RdRp) [129] – results in increased antigen production compared to non‐replicating RNA [130]. This in turn drives stronger priming of CD8^+^ T cell responses [130]. Nucleoside modification of the mRNA construct—where uracil bases are modified to avoid triggering pattern recognition receptors (reviewed in [17])—also results in increased protein production [131, 132, 133], presumably due to increased persistence of the mRNA molecule. Thus, the mRNA molecule can be engineered to directly regulate antigen levels.

**Figure 2:**
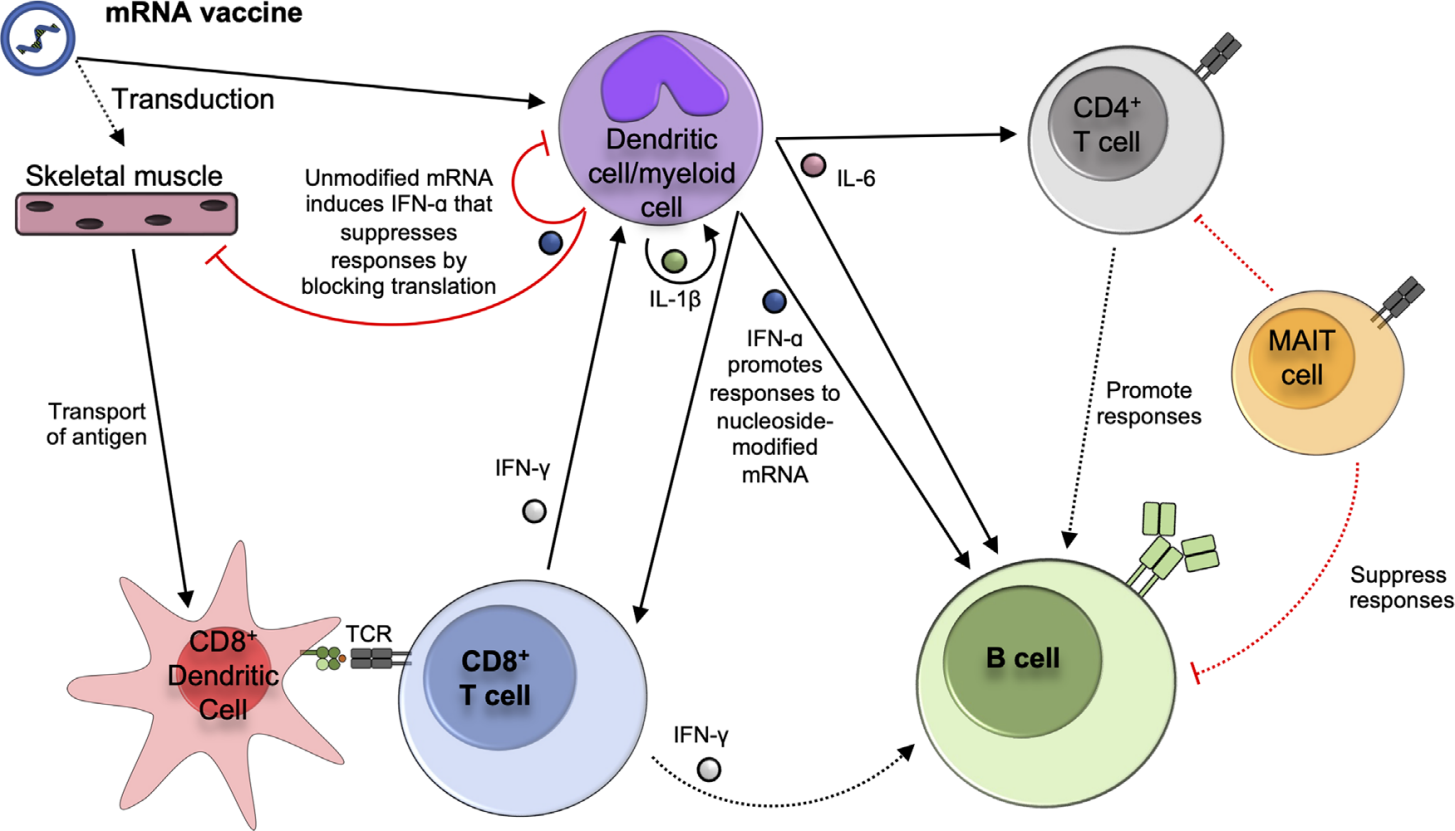
**Pathways known to promote or inhibit the induction of cellular and humoral immune responses following mRNA vaccine immunization**. Key pathways and immune processes identified by mouse studies are shown in solid lines. Black arrows denote processes shown to promote immune responses, while red lines denote inhibition. Dashed lines indicate where correlative data in humans or non‐human primates suggests an interaction between cell types or the mouse studies have identified a cytokine as important but the exact producer and target cell are unknown. Where cells have been shown to interact, but molecular mechanisms have not been determined, this is noted. Not all pathways have been examined for all different types of mRNA vaccines. The presented model is focused on data specifically in the context of nucleoside‐modified mRNA vaccines, except where specifically noted. See text for references for indicated pathways.

Unlike Ad vectors, which primarily transduce local tissues following intramuscular immunization, mRNA vaccines can display broad biodistribution [134]. Altering the chemical and physical properties of the LNP (e.g. size, charge, and acidity) through manipulating the lipids used can promote specific targeting of tissues and cell types, and thereby alter immunogenicity based on route of delivery [134, 135]. One study examining BNT162b2‐vaccinated humans found spike protein‐containing exosomes in the plasma for several weeks following immunization [136]. Following intramuscular immunization of mice with BNT162b2, antigen was readily detected for several days in the serum and could be found at low levels in the spleen [137]. Interestingly negligible antigen was detected within the muscle, which stands in strong contrast to the major depot of antigen at the injection site following Ad vector immunization (Fig. 2) [22, 23, 24]. However, the systemic spread of the vaccine and/or antigen following immunization has an unclear role in the immunogenicity of these vaccines.

Despite detectable antigen in circulation and in tissues far from the site of injection, the draining LN was the primary site of antigen production and non‐draining lymph nodes did not appear to contribute [137]. A series of elegant studies examined axillary LN responses in humans following BNT162b2 or mRNA‐1273 vaccination using fine needle aspiration or core biopsies clearly identified the local draining LN as the primary site of germinal center B cell responses and an accumulation of spike‐specific CD4^+^ T_FH_ cells [138, 139, 140, 141, 142]. Although sampling of non‐draining LNs was limited, there was a clear focus of immune induction within the draining LN as compared to other sites. Interestingly, despite readily available antigen within the LN, direct transduction of DCs does not appear sufficient for priming of T cell responses as cross‐presenting CD8^+^ DCs were still found to be critical (Fig. 2) [137]. Following intradermal immunization, a redundant role for Langerhans cells and CD8^+^ DCs was identified for the induction of antibody and T_FH_ cell responses [143]. CD8^+^ T cell responses were not measured. However, even in the absence of these two DC populations, vaccinated mice were still protected from influenza or SARS‐CoV‐2 infection, suggesting these populations play only a minor role in induction of humoral immunity.

### Type I interferons and regulation of cellular and humoral immunity

The in vitro transcribed RNA used for mRNA‐LNP vaccines has potent capacity to induce type I interferon production [144, 145, 146]. This is due to both TLR7‐mediated sensing of the mRNA molecules [147, 148] and detection of dsRNA that is produced as a byproduct of in vitro transcription [149]. For both unmodified mRNA vaccines and saRNA vaccines, several studies found that the induction of type I interferon dampens the priming of both antibody and T cell responses (Fig. 2) [144, 145, 146]. This impairment is likely due to IFN‐mediated clearance of the mRNA molecules, thereby attenuating duration of antigen expression (as discussed above). In contrast, one study found control of tumor growth by repeated therapeutic immunization with an unmodified mRNA vaccine required type I interferon signaling, which was associated with T cell functionality [135].

Given the apparent detrimental effect of interferon induction on protein production, considerable efforts have been made to engineer mRNA molecules to avoid TLR sensing and thereby prevent induction of type I interferon, particularly in the context of the gene therapy field [17]. Nucleoside modification by replacing uracil bases with either pseudouridine (Ψ) or 5‐methyl uridine (m5U) dampens TLR sensing [150]. Furthermore, altering in vitro synthesis production methods to remove dsRNA contaminants further dampens TLR sensing and induction of interferons [151]. Despite nucleoside modification being initially targeted toward generating non‐stimulatory mRNA, this approach has been surprisingly effective in the context of vaccination (as evidenced by the BNT162b2 and mRNA‐1273 vaccines).

As nucleoside modification effectively dampens induction of type I interferon in vitro and in vivo [151, 152], it was reasonably assumed that nucleoside‐modified mRNA vaccines functioned in a manner independent of type I interferon signaling. However, transcriptomic analysis at early timepoints following vaccination with BNT162b2 did identify clear signatures of type I interferon following immunization of both humans [153] and mice [137]. Using *Ifnar1^−/−^* mice, the authors went on to demonstrate that absence of type I interferon resulted in a substantially impaired CD8^+^ T cell response with modest reductions in antibody titers (Fig. 2). The authors identified the cGAS‐STING pathway as the mechanism of type I interferon induction. Thus, suggesting that possibly tissue damage associated with the vaccines instead of direct sensing of vaccine RNA was responsible for the interferon. The cells responsible for production of type I interferon in this context remains to be determined.

It remains unclear how important nucleoside‐modification is for a successful human mRNA vaccine. Preclinical comparison of unmodified versus m1Ψ‐modified mRNA vaccines have generated conflicting results on relative immunogenicity of the two approaches [133, 154]. One study found significantly enhanced antibody, B cell, CD4^+^ T cell, and CD8^+^ T cell responses when using an m1Ψ‐modified mRNA vaccine [133]. While the other study, which only examined CD8^+^ T cell responses, found the opposite [154]. The candidate COVID‐19 unmodified mRNA vaccine trialed by CureVac had promising Phase 1 immunogenicity data [155], and Phase 2/3 trial data suggested efficacy of 70% against moderate‐to‐severe COVID‐19 [19]. However, the candidate was not pursued further in part due to logistical considerations, which complicates comparison to the licensed nucleoside‐modified vaccines. BioNTech's candidate mRNA vaccine platform for cancer, which uses unmodified mRNA, has shown promising immunogenicity in a Phase 1 trial [156], but a comparison to a similar nucleoside‐modified construct has not been made.

An early saRNA COVID‐19 vaccine candidate had disappointing immunogenicity in a Phase 1 trial [20], despite promising mouse data [157]. However, a recent press release from Arcturus Therapeutics on a Phase III efficacy trial of their saRNA vaccine against COVID‐19 reported 95.3% (95% CI: 80.4% to 98.9%) efficacy against severe disease (including death) [158]. These results are similar to both the phase III trial results and real‐world efficacy numbers (during the contemporaneous waves of the Delta and Omicron variants) reported with the BNT162b2 and mRNA‐1273 vaccines at a similar time post‐immunization [159, 160, 161]. It should be noted, we await release of the peer‐reviewed results of Arcturus Therapeutic's trial. Regardless, these data suggest that saRNA vaccines may also be effective in humans.

### Regulation of immunogenicity by inflammatory cytokines

The lipid portion of an mRNA‐LNP vaccine also plays a role in stimulating the innate immune response. IL‐6 produced by the LNP promotes the differentiation of T_FH_ cells and thereby enhances B cell and antibody responses (Fig. 2) [143, 162]. Interestingly, the LNP could enhance antibody responses even when co‐delivered with recombinant protein (without encapsulation) [162]. One study found that mice lacking T_FH_ cells (*Bcl6^fl/fl^* x *CD4^Cre^*) had only partially impaired antibody responses as measured by titer, neutralization capacity, and somatic hypermutation following immunization with BNT162b2 or mRNA‐1273 [163], raising the prospect that T cell‐independent antibody responses might be raised by these vaccines (Fig. 2). This needs to be investigated in greater detail.

In addition to IL‐6, the LNP strongly induces production of IL‐1β (Fig. 2) [164, 165]. The presence of RNA and sensing of it (ie. no nucleoside modification) is necessary for induction of IL‐1β [165], likely through priming the inflammasome [166]. IL‐1β was also found to be a key upstream cytokine for several LNP‐induced cytokines, including IL‐6 and IFN‐ɑ (Fig. 2). This study was performed primarily using unmodified mRNA‐based vaccine formulations, likely contributing to the strong IFN‐ɑ induction. Intranasal immunization of mice with an experimental mRNA‐LNP vaccine using the LNP produced by Acuitas Therapeutics – the lipid used in the BNT162b2 vaccine – resulted in fatal inflammation in a dose‐dependent manner [164]. This appears to be a specific characteristic of this lipid formulation, as other mRNA‐LNP constructs have been successfully administered to the lungs of mice [167, 168].

An interesting study found that MAIT cell frequencies at baseline or two weeks after the second dose of the BNT162b2 vaccine were positively correlated with vaccine‐induced CD4^+^ T cell and antibody responses [169]. However, despite the vaccine not altering levels of ex vivo MAIT activation at the timepoints sampled, the degree of MAIT cell activation was inversely associated with vaccine‐induced CD4^+^ T cell and antibody responses. Whether this reflects a direct role for MAIT cells in dampening mRNA vaccine immunogenicity, as seen with adenovirus vectors (discussed above), remains to be determined (Fig. 2).

Several studies have reported increased innate inflammatory cytokine responses following the second dose of BNT162b2 as compared to the first in both humans and mice [137, 153, 170]. This coincides with the increased adverse events reported following second vaccine dose [160]. Interferon‐γ was the most strongly induced cytokine following the second vaccine dose. In the mouse model, T cells (both CD8^+^ and CD4^+^) and NK cells were the major source of IFN‐γ [137]. In vaccinated humans, post‐boost IFN‐γ was correlated with increased activation of myeloid cells (Fig. 2) [153], and a mechanistic association between post‐boost IFN‐γ and activation of innate activation was confirmed by blocking experiments in mice [137]. Another study found a correlation between IFN‐γ levels and post‐boost antibody titers in a human cohort (Fig. 2) [170]. However, a causal relationship was not demonstrated in the mouse model, nor were CD8^+^ T cell responses significantly altered [137]. Thus, the exact impact this enhanced inflammatory cytokine response has on the rest of the immune response remains to be resolved.

## Conclusions

As a result of the COVID‐19 pandemic, adenovirus vectors and mRNA vaccines have recently fulfilled their promise as highly manipulable, immunogenic, and efficacious vaccine platforms. For most of their development history, research using these platforms has, understandably, focused on improving and refining the immunogenicity of these constructs, often in a very empirical manner. This has necessarily led to an abundance of candidate vaccine platforms all with slightly differing biology, which has complicated efforts to understand the fundamental immunologic mechanisms governing the immunogenicity (or lack thereof) of these vaccine constructs. With the licensure and widespread use of multiple Ad vector‐based vaccines and two nucleoside‐modified mRNA‐LNP vaccines, there is a clear incentive to better understand the biology underpinning these technologies. The years ahead should be filled with major advances as we elucidate mechanistically how these vaccines interact with the immune system.

## Conflict of interest

P.K. is a named inventor on a patent application in the field of cancer vaccines. N.P. declares no commercial or financial conflict of interest.

## Author contributions

N.M.P. and P.K. conceived and wrote the review.

## Funding

P.K. (Wellcome [222426/Z/21/Z], NIHR Senior Investigator).

## Data Availability

Data sharing not applicable to this article as no datasets were generated or analyzed during the current study.
